# Necroptotic and Apoptotic Pathways in Sepsis: A Comparative Analysis of Pediatric and Adult ICU Patients

**DOI:** 10.3390/biomedicines13071747

**Published:** 2025-07-17

**Authors:** George Briassoulis, Konstantina Tzermia, Kalliopi Bastaki, Marianna Miliaraki, Panagiotis Briassoulis, Athina Damianaki, Eumorfia Kondili, Stavroula Ilia

**Affiliations:** 1 Postgraduate Program “Emergency and Intensive Care in Children, Adolescents and Young Adults”, School of Medicine, University of Crete, 71003 Heraklion, Greece; tzermiak@gmail.com (K.T.); pbastaki@gmail.com (K.B.); mmiliaraki@uoc.gr (M.M.); briaspan@med.uoa.gr (P.B.); damianak@uoc.gr (A.D.); kondylie@uoc.gr (E.K.); stavroula.ilia@uoc.gr (S.I.); 2 Pediatric Intensive Care Unit, University Hospital, School of Medicine, University of Crete, 71110 Heraklion, Greece; 3 Second Department of Anaesthesiology, Attikon University Hospital, School of Medicine, National and Kapodistrian University of Athens, 12461 Athens, Greece; 4 Intensive Care Unit, University Hospital, School of Medicine, University of Crete, 71110 Heraklion, Greece

**Keywords:** sepsis, necroptosis, caspase-8, RIPK1, IL-18, A20, pediatric, ICU, biomarkers, cell death, inflammation

## Abstract

**Background:** Necroptosis, a regulated form of inflammatory cell death, is increasingly recognized as a key driver of sepsis and critical illness. The balance between necroptosis and apoptosis may influence immune responses and outcomes in ICU patients. **Objective:** To evaluate necroptosis- and apoptosis-related protein expression in critically ill pediatric and adult patients with sepsis/septic shock, trauma/SIRS, or cardiac conditions, and assess their association with clinical outcomes. **Methods:** In this prospective, observational study, 88 patients admitted to a tertiary ICU were categorized into four groups: sepsis/septic shock, trauma/SIRS, cardiac disease, and healthy controls. Serum levels of RIPK1, RIPK3, MLKL, A20, caspase-8, IL-1β, and IL-18 were measured within 24 h of admission using ELISA. Biomarkers were analyzed by disease group, age, and severity indices. **Results:** Patients with sepsis—both adults and children—exhibited significantly elevated levels of RIPK1, IL-1β, and IL-18 (*p* < 0.001) and reduced levels of caspase-8 (*p* = 0.015), indicating activation of the necroptosis pathway. A20 was significantly upregulated (*p* < 0.001) and independently associated with lactate levels. RIPK1, IL-1β, and IL-18 were positively correlated with ICU length of stay and illness severity, whereas caspase-8 showed an inverse correlation. ROC analysis demonstrated strong predictive performance for sepsis/septic shock using RIPK1 (AUC = 0.81), IL-18 (AUC = 0.71), and A20 (AUC = 0.71); conversely, caspase-8 was inversely associated with sepsis (AUC = 0.32). **Conclusions:** Necroptosis appears to play a central role in the pathophysiology of sepsis across age groups. Elevated levels of RIPK1, IL-1β, IL-18, and A20 may serve as biomarkers of disease severity, while reduced caspase-8 supports a shift away from apoptosis toward necroptotic cell death. These findings highlight the potential of necroptosis-related pathways as targets for risk stratification and therapeutic intervention in critically ill patients of all ages.

## 1. Introduction

Sepsis and systemic inflammatory response syndrome (SIRS) remain leading causes of morbidity and mortality among pediatric and adult patients admitted to intensive care units (ICUs) worldwide. Despite advances in critical care, the underlying mechanisms driving organ dysfunction in these conditions are not fully understood, limiting the development of targeted therapies.

Necroptosis, a regulated form of necrotic cell death, has emerged as a significant contributor to the pathophysiology of various inflammatory diseases, including sepsis, trauma-induced SIRS, and cardiac conditions. Unlike apoptosis, necroptosis is characterized by the activation of receptor-interacting protein kinases (RIPK)1 and RIPK3, leading to the phosphorylation of mixed lineage kinase domain-like protein (MLKL), which results in cell membrane rupture and the release of pro-inflammatory intracellular contents [1,2,3,4]. This process exacerbates systemic inflammation and contributes to multiorgan failure.

In pediatric populations, the role of necroptosis in critical illnesses is increasingly recognized. Experimental studies have shown that pharmacological inhibition of necroptosis can attenuate lung injury and improve survival in neonatal models of sepsis [3]. Similarly, necroptosis has been implicated in the pathogenesis of traumatic brain injury and acute cardiac conditions—common causes of PICU admissions [5,6]. These findings suggest that targeting necroptotic signaling may represent a novel therapeutic approach in both adult and pediatric intensive care settings.

In this context, specific proteins such as A20, a ubiquitin-editing enzyme, play a central role in modulating inflammation and cell death by inhibiting RIPK3-dependent necroptosis [7]. Conversely, caspase-8 is a pivotal mediator of apoptosis that also acts as an endogenous inhibitor of necroptosis through cleavage of RIPK1 and RIPK3, preventing the execution of the necroptotic cascade [3,6,8,9]. Dysregulation of either protein has been implicated in heightened inflammatory responses and poor outcomes in sepsis [10,11].

Understanding the balance between necroptosis and apoptosis in sepsis is crucial, as it may unveil novel biomarkers for disease severity and potential therapeutic targets [12,13,14,15]. This study aims to elucidate the expression patterns of key necroptotic and apoptotic proteins—including RIPK1, RIPK3, MLKL, A20, caspase-8, interleukin (IL)-1β, and IL-18—in critically ill pediatric and adult patients with sepsis, and to explore their correlation with clinical severity indices and patient outcomes, in comparison to controls.

## 2. Materials and Methods

### 2.1. Study Design

This is a prospective, single-center, observational study conducted at the University General Hospital of Heraklion, encompassing the Intensive Care Unit (ICU), Pediatric Intensive Care Unit (PICU), and Cardiac Intensive Care Unit (CICU). The primary objective was to assess the expression of necroptosis-related proteins in critically ill pediatric and adult patients with sepsis, trauma/surgery-induced SIRS, or heart disease and to compare findings with those from healthy control subjects. The study also aimed to investigate the relationships between necroptosis protein levels and clinical or laboratory severity indices, as well as patient outcomes. This study adhered to STROBE reporting guidelines for observational research.

### 2.2. Study Population

The study enrolled critically ill pediatric and adult patients admitted between June and November 2023. Patients were prospectively categorized into four groups: Sepsis/septic shock (n = 23): patients fulfilling the Sepsis-3 criteria; SIRS (n = 29): patients with non-infectious SIRS due to surgery or trauma; cardiac (n = 19): patients monitored in the CICU for acute heart conditions; and healthy controls (n = 17): age-matched pediatric and adult subjects without known acute or chronic diseases.

Inclusion criteria comprised admission to the ICU, PICU, or CICU with a confirmed diagnosis of sepsis, trauma/SIRS, or acute cardiac disease. Exclusion criteria were the presence of chronic illness (e.g., autoimmune disease, diabetes, and renal or hepatic insufficiency), congenital or acquired immunodeficiencies, malignancies, or current use of immunosuppressive or immunomodulatory therapy.

### 2.3. Data Collection

Clinical and laboratory data were obtained from the patients’ official medical records (both handwritten and electronic). Documentation included the following: diagnostic categorization according to international criteria (sepsis, SIRS, or cardiac); disease severity scores: SOFA (Sequential Organ Failure Assessment), qSOFA, APACHE II (Acute Physiology and Chronic Health Evaluation), PRISM III (Pediatric Risk of Mortality), and PeLOD (Pediatric Logistic Organ Dysfunction), as applicable by age group; and laboratory markers of systemic inflammation and infection (e.g., CRP, procalcitonin, and leukocyte count).

### 2.4. Ethics and Consent

This study was approved by the Administrative Board, the Scientific Council of the University General Hospital of Heraklion, and the Bioethics Committee (Approval No. 19050/2023; Date: 26 June 2023). All procedures complied with the principles of the Declaration of Helsinki and the General Data Protection Regulation (GDPR, EU 2016/679). Given the prospective nature of the study and the collection of sensitive clinical and biological data, written informed consent was obtained from all participants or their legal guardians, as required by institutional and legal regulations.

### 2.5. Laboratory Methods

Peripheral blood samples were collected within the first 24 h following ICU admission and clinical confirmation of sepsis or SIRS. Serum was separated and stored under standardized conditions until analysis.

Quantification of necroptosis-associated proteins—RIPK1, RIPK3, MLKL, and A20—as well as caspase-8 and proinflammatory cytokines IL-1β and IL-18, was performed using commercially available sandwich ELISA kits (ELK Biotechnology, Denver, CO, USA), according to the manufacturer’s instructions. The assays were conducted in duplicate, and optical density was measured at 450 nm using a microplate reader. Protein concentrations were derived from standard curves based on known calibrator concentrations.

Detection limits were as follows: RIPK1 (0.063 ng/mL), RIPK3 and MLKL (0.122 ng/mL), A20 (0.17 ng/mL), caspase-8 (0.244 ng/mL), IL-18 (5.9 pg/mL), and IL-1β (5.8 pg/mL). These biomarker levels were analyzed in relation to clinical severity scores (SOFA, qSOFA, APACHE II, PRISM, and PELOD-2) and outcomes and were compared across diagnostic groups and healthy controls.

### 2.6. Rationale for Marker Selection

The biomarkers included in this study were selected to represent the central regulators of necroptosis and apoptosis and to capture key mediators of necroinflammatory signaling in sepsis [16].

- RIPK1, RIPK3, MLKL, and A20 are core components of the necroptosis cascade [3]: RIPK1 initiates necrosome formation via RIPK3 phosphorylation, MLKL executes membrane disruption, and A20 modulates pathway activity by limiting ubiquitination events—and thus inflammatory cell death—during sepsis.
- Caspase-8 was chosen as the apoptosis-related marker due to its dual functionality: It triggers extrinsic apoptosis through FADD activation and simultaneously suppresses necroptosis by cleaving RIPK1 and RIPK3, thus preventing necrosome formation [5]. Therefore, caspase-8 serves as a pivotal “molecular switch” at the intersection of apoptotic and necroptotic signaling [6].
- While caspase-3 is a downstream executioner of apoptosis, it does not reflect the regulatory balance between apoptotic and necroptotic pathways and was therefore not included in this phase of the study [17].
- We included IL-1β and IL-18 because these cytokines are products of inflammasome activation and serve as biomarkers of necroinflammation and pyroptosis—pathways that intersect cytokine-mediated inflammation and programmed necrosis [9]. Experimental models demonstrate that combined inhibition of IL-1β and IL-18 protects against lethal sepsis, underscoring their relevance as downstream effectors of cell–death–induced inflammation. Elevated IL-18 levels, in particular, have been independently associated with sepsis severity and mortality [18].
- In contrast, classical pro-inflammatory cytokines such as TNF-α and IL-6 were excluded from our biomarker panel due to their non-specificity, rapid kinetics, and broad elevation in various inflammatory conditions (e.g., trauma, surgery, and non-septic illness), which limit their usefulness as stable indicators of programmed cell death pathways in critically ill patients [19].

By focusing on these markers, in our study, we aimed to dissect the molecular interplay between necroptosis, apoptosis, and necroinflammation during critical illness and sepsis, rather than measuring systemic inflammation signals with limited mechanistic specificity.

### 2.7. Statistical Analysis

Data were recorded in Microsoft Excel and analyzed using SPSS software (version 29.0, IBM Corp., Armonk, NY, USA). Continuous variables were tested for normality and are presented as the mean ± standard deviation (SD) or median with interquartile range (IQR), as appropriate. Categorical variables were expressed as absolute (n) and relative (%) frequencies. Group comparisons for continuous variables were performed using t-tests or one-way ANOVA for normally distributed data and Mann–Whitney U or Kruskal–Wallis tests for non-normally distributed data. Categorical variables were compared using the chi-square test or Fisher’s exact test. Spearman’s rank correlation coefficient was used to assess associations between continuous variables. Backwards stepwise multivariate linear regression was performed to identify independent associations between biomarkers and clinical outcomes. Receiver operating characteristic (ROC) curve analyses were conducted to evaluate the predictive performance of necroptosis biomarkers. A *p*-value < 0.05 was considered statistically significant.

## 3. Results

### 3.1. Demographic and Clinical Characteristics

This prospective, single-center clinical study included 88 participants admitted to the Pediatric and Adult Intensive Care Units (ICUs) and the Cardiac Intensive Care Unit at the University Hospital of Heraklion between June and November 2023. The cohort comprised 56 adults (63.6%) and 32 children (36.4%), categorized into four groups: sepsis (26.1%), trauma/surgery (SIRS) (33%), cardiac conditions (21.6%), and healthy controls (19.3%).

Significant differences were observed between adults and children in age, body weight, and Body Mass Index (BMI) (*p* < 0.001). Undernutrition was more prevalent among children (18.8%) compared to adults (3.6%), whereas overweight status was more common in adults (26.8%) than in children (9.4%) (*p* = 0.02). Adults exhibited higher rates of comorbidities (51.8% vs. 6.3%, *p* < 0.001) and required vasopressor support more frequently (51.8% vs. 6.3%, *p* < 0.001). No significant differences were observed between the groups in terms of gender distribution, disease severity scores, mechanical ventilation, ICU length of stay, or mortality rates. Detailed demographic and clinical data are presented in Table 1, Table 2 and Table 3.

### 3.2. Laboratory Findings

Laboratory analyses revealed that adults had significantly higher levels of glucose (152 vs. 103 mg/dL, *p* < 0.001), urea (49 vs. 20 mg/dL, *p* < 0.001), and bilirubin (1.2 vs. 0.5 mg/dL, *p* = 0.008) compared to pediatric patients. Other laboratory parameters did not show significant differences between the two groups. Comprehensive laboratory results are detailed in Table S1.

### 3.3. Necroptosis Biomarkers

Among ICU patients, RIPK-1 levels were significantly higher in adults (median [IQR]: 20.2 [7.4–46.5] ng/mL) compared to children (6.2 [4.4–16.2] ng/mL; *p* = 0.021). Notably, two adult patients—one with septic shock (106 ng/mL) and one with traumatic liver rupture (73 ng/mL)—exhibited markedly elevated values that contributed to the higher median RIPK-1 levels in the adult group. In contrast, caspase-8 levels were significantly elevated in children (19.1 [14–28] ng/mL) relative to adults (13.8 [11–20] ng/mL; *p* = 0.008). No statistically significant differences were observed between adult and pediatric ICU patients in the levels of RIPK-3, MLKL, A20, IL-1β, or IL-18 (Table S1).

In the healthy control population, RIPK-1 levels did not significantly differ between adults (3.17 ng/mL) and children (2.47 ng/mL; *p* = 0.009). Similarly, caspase-8 levels were comparable in pediatric (24.7 ng/mL) and adult (24.6 ng/mL) controls (*p* = 0.006). As with ICU patients, no significant age-related differences were found in the remaining necroptosis or inflammasome-related markers among healthy individuals.

### 3.4. Biomarker Distribution Across Clinical Groups

Comparative analysis across diagnostic categories revealed that patients with sepsis exhibited significantly elevated levels of RIPK-1, IL-1β, and IL-18, along with reduced caspase-8 levels, relative to patients with SIRS or cardiac conditions. Importantly, the exclusion of two adult outliers—one from the sepsis group and one from the SIRS group—did not alter the statistical significance of these intergroup differences.

Although the overall sample size limited power for detailed subgroup analyses, particularly within pediatric diagnostic strata, we conducted additional stratified comparisons. Specifically, age-stratified analyses of diagnostic group differences are presented in Table S2, demonstrating consistent trends between adult and pediatric cohorts. Given the absence of meaningful age-related effect modification, biomarker data were pooled for the primary analysis to enhance statistical robustness. To improve interpretability, comprehensive comparisons across diagnostic groups are summarized in Table 4, where group sizes were sufficiently comparable to support meaningful statistical inference.

### 3.5. Expression of Necroptosis Pathway Proteins

**Figure 1:**
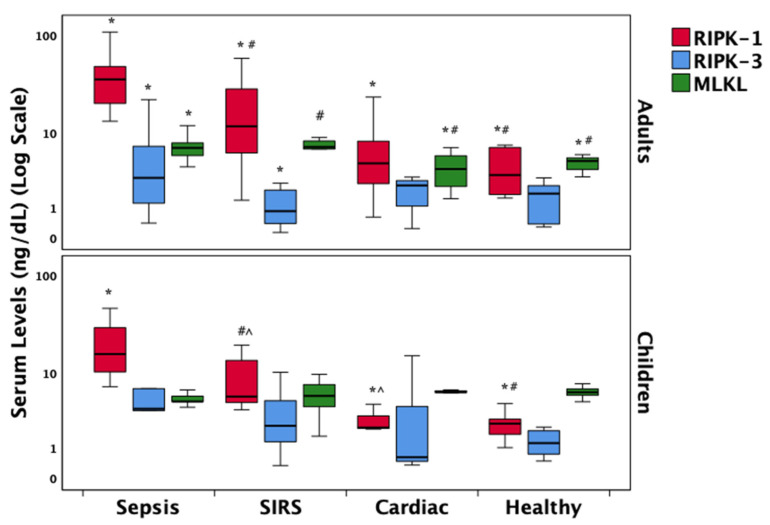
Comparative distribution of key necroptosis-related protein markers across diagnostic groups. Box plots display group medians (bold black lines), interquartile ranges (25th to 75th percentiles as box boundaries), and non-outlier minimum and maximum values (whiskers). Statistically significant differences (*p* < 0.05) between diagnostic groups are denoted relative to sepsis (*), SIRS (#), or cardiac patients (^) using post hoc Dunn’s pairwise tests with Bonferroni correction. Abbreviations: SIRS = Systemic Inflammatory Response Syndrome; RIPK = Receptor-Interacting Protein Kinase; MLKL = Mixed Lineage Kinase Domain-Like Protein.

RIPK-1 and RIPK-3, key mediators of the necroptosis pathway, were elevated in patients with sepsis across both age groups, with RIPK-1 reaching statistical significance (*p* < 0.001). Among adults, patients with SIRS also demonstrated significantly higher RIPK-1 levels compared to those with cardiac conditions and healthy controls (Table S2). RIPK-3 followed a similar pattern, showing increased levels in the sepsis group relative to SIRS in adults and to cardiac patients in children (*p* = 0.06). MLKL concentrations were elevated in adult patients with sepsis and SIRS compared to other groups; however, no significant group-wise differences in MLKL levels were observed in the pediatric cohort. These distributions are visually summarized in Figure 1.

### 3.6. Inflammatory Mediators of Necroptosis

**Figure 2:**
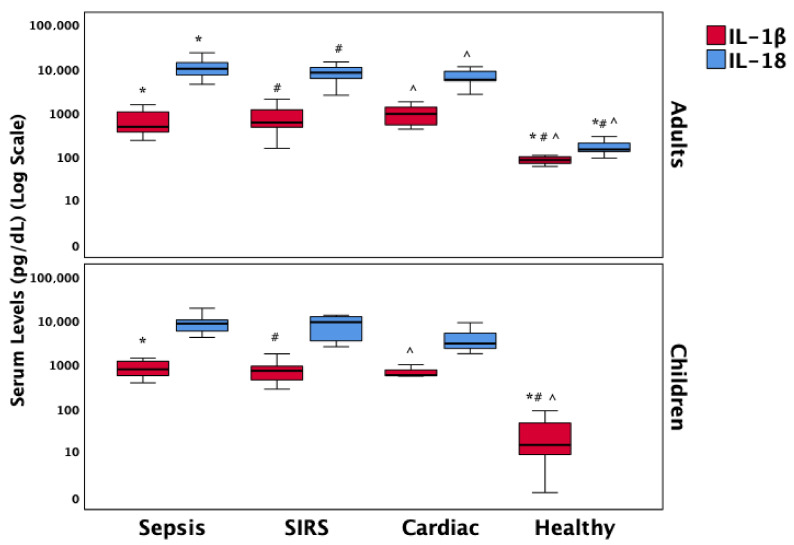
Comparative distribution of the pro-inflammatory necroptosis mediators IL-1β and IL-18 across diagnostic groups in adult and pediatric patients. Box plots represent group medians (bold black lines), interquartile ranges (25th–75th percentiles), and whiskers showing the minimum and maximum non-outlier values. Statistically significant differences (*p* < 0.05) between diagnostic groups are indicated relative to sepsis (*), SIRS (#), or cardiac patients (^) based on post hoc Dunn’s pairwise tests with Bonferroni correction. Abbreviations: SIRS = Systemic Inflammatory Response Syndrome; IL = Interleukin.

IL-1β levels were significantly elevated in all three patient groups—sepsis, SIRS, and cardiac—compared to healthy controls, with similar distributions observed in both adults and children. IL-18 levels followed a comparable pattern among the patient groups, showing significantly higher concentrations in adults relative to healthy controls. However, due to missing data for IL-18 in the pediatric healthy control group, direct comparison in children was not feasible. These biomarker patterns are illustrated in Figure 2.

### 3.7. Regulatory Proteins in the Necroptosis Pathway

**Figure 3:**
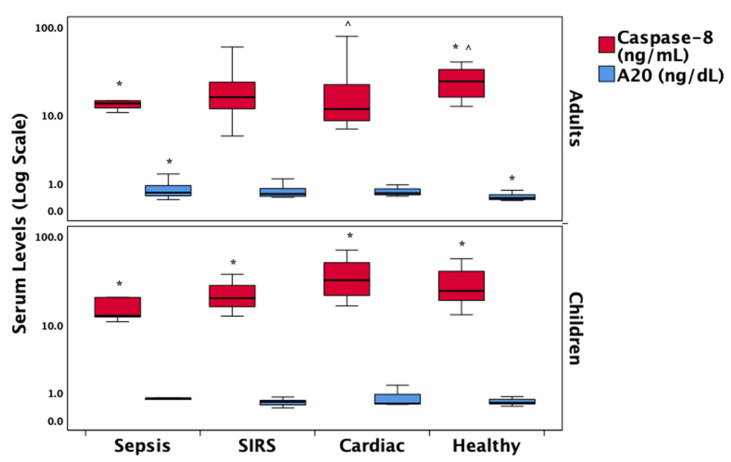
Comparative distribution of A20, a ubiquitin-editing enzyme involved in the regulation of cell death pathways, and caspase-8, a key apoptosis-promoting and necroptosis-inhibiting protein, across diagnostic groups in adult and pediatric patients. Box plots show group medians (bold black lines), interquartile ranges (25th–75th percentiles), and whiskers representing the minimum and maximum non-outlier values. Statistically significant differences (*p* < 0.05) between diagnostic groups are indicated relative to sepsis (*) or cardiac patients (^) based on post hoc Dunn’s pairwise tests with Bonferroni correction. Abbreviations: SIRS = Systemic Inflammatory Response Syndrome.

Caspase-8 levels were significantly reduced in the sepsis group compared to healthy controls and cardiac patients in both adults and children. The ubiquitin-editing enzyme A20 was significantly elevated in adult sepsis patients compared to healthy controls. However, due to missing data in pediatric sepsis cases, reliable analysis of A20 levels in this subgroup was not feasible. These findings are illustrated in Figure 3.

### 3.8. Correlations with Clinical Outcomes

RIPK1 levels demonstrated significant positive correlations with APACHE II (rs = 0.39, *p* = 0.003), SOFA (rs = 0.35, *p* = 0.030), and PRISM scores (rs = 0.46, *p* = 0.030), suggesting a link with overall illness severity. Additionally, IL-1β correlated with PRISM (rs = 0.43, *p* = 0.040), and IL-18 showed a strong association with SOFA (rs = 0.44, *p* < 0.001), further supporting their relevance as markers of disease burden. Significant positive correlations were found between ICU length of stay and levels of RIPK-1 (rs = 0.5, *p* < 0.001), IL-1β (rs = 0.43, *p* < 0.001), and IL-18 (rs = 0.48, *p* < 0.001). Caspase-8 levels were negatively correlated with ICU stay duration (rs = −0.24, *p* = 0.028). The positive correlation between RIPK-1 and ICU stay was consistent across both adult and pediatric patients. The negative correlation with caspase-8 was particularly evident in sepsis and SIRS groups, as shown in Figure S1.

### 3.9. Independent Associations

A multivariate linear regression analysis, adjusted for age groups, identified A20 as the sole biomarker independently associated with blood lactate levels (Standardized Coefficient Beta = 0.55, *p* < 0.001).

### 3.10. Predictive Value of Necroptosis Biomarkers

**Figure 4:**
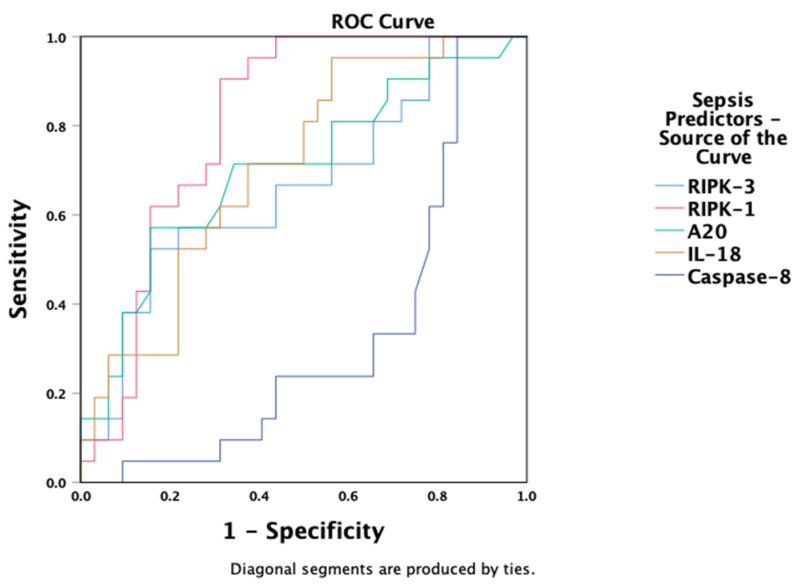
ROC analysis of early (within 24 h) necroptosis biomarker activation in ICU patients with sepsis or septic shock. Abbreviations: RIPK = Receptor-Interacting Protein Kinase; IL = Interleukin. ROC = Receiver operating characteristic.

Receiver Operating Characteristic analysis demonstrated that RIPK-1 (AUC = 0.81, 95% CI: 0.69–0.93, *p* < 0.001), IL-18 (AUC = 0.71, 95% CI: 0.57–0.85, *p* < 0.01), and A20 (AUC = 0.71, 95% CI: 0.56–0.85, *p* = 0.012) were effective predictors of sepsis or septic shock diagnosis within the first 24 h of ICU admission (Figure 4). RIPK-3 showed moderate predictive value (AUC = 0.66, 95% CI: 0.51–0.82, *p* = 0.044), while caspase-8 demonstrated a negative association (AUC = 0.32, 95% CI: 0.17–0.47, *p* = 0.029).

## 4. Discussion

This prospective study provides novel insights into the expression of necroptosis- and apoptosis-related proteins in critically ill pediatric and adult patients with sepsis, trauma/SIRS, or cardiac disease. We observed prominent necroptosis activation in sepsis—evidenced by elevated RIPK-1, IL-1β, and IL-18 levels and suppressed caspase-8 levels. These biomarker changes correlated with prolonged ICU stay and higher illness severity, underscoring the mechanistic relevance of necroptosis in systemic inflammation and organ dysfunction.

### 4.1. Necroptosis Activation in Sepsis

A particularly striking finding is the marked increase in RIPK-1 levels among septic patients—both adults and children—relative to all other diagnostic groups. As a central mediator of necroptosis, RIPK-1 facilitates inflammatory cell death, especially when caspase-8 activity is diminished [20,21]. The concurrent elevation of RIPK-3, particularly in adult sepsis patients, although not statistically significant in children, aligns with animal and experimental data indicating that activation of RIPK(1/3) exacerbates tissue injury [22,23,24], cytokine release, and necroptotic cell death in sepsis models [5,25].

Equally significant is the consistent suppression of caspase-8 in both adult and pediatric sepsis cohorts when compared to healthy controls. Caspase-8 plays a critical inhibitory role over RIPK-1-RIPK3 complex formation; thus, its downregulation removes a key checkpoint, enabling necrosome assembly and pro-inflammatory cell death [6,26,27]. In our cohort, caspase-8 levels correlated inversely with ICU length of stay—equivalently in both age groups—which further supports its functional and prognostic relevance.

### 4.2. The Role of Pro-Inflammatory Cytokines IL-1β and IL-18

Our study also demonstrated substantially elevated levels of IL-1β and IL-18 in critically ill patients—including those with sepsis—compared to healthy adult and pediatric controls. Notably, IL-18 exhibited the highest discriminative performance in our ROC analysis, suggesting strong diagnostic value. These cytokines are key products of inflammasome activation and serve as potent effectors of necroinflammation, often released during membrane rupture inherent in necroptotic or pyroptotic cell death pathways [28,29,30]. Elevated IL-18 levels have been consistently associated with sepsis severity and poorer outcomes in critically ill patients [18], outperforming conventional markers like procalcitonin and CRP in diagnostic utility in some studies [31,32,33]. Similarly, IL-1β is recognized as a central instigator of the cytokine storm, amplifying inflammatory cascades and correlating strongly with sepsis severity and prognosis [34,35].

The high expression of IL-1β and IL-18 in our septic cohort further supports the involvement of necroptotic and inflammasome-mediated mechanisms in critical illness [36]. Importantly, these cytokines were also elevated in SIRS and cardiac patients across both age groups—compared to controls—indicating that sterile inflammation and cardiac stress can provoke necroinflammatory signaling even in non-infectious conditions. This pattern aligns with prior evidence implicating necroptosis and inflammasome activation in myocardial infarction and non-infectious surgical trauma models [37,38].

### 4.3. A20 as a Regulatory Checkpoint

In our study, A20 (TNFAIP3)—a ubiquitin-editing enzyme and known negative regulator of necroptosis and NF-κB signaling—was significantly upregulated in adult sepsis patients compared to healthy controls. Notably, higher A20 expression correlated independently with elevated blood lactate levels, a surrogate marker of tissue hypoxia and illness severity.

A20 is a well-documented regulator of programmed cell death: by removing K63-linked ubiquitin chains from RIPK1, A20 prevents the formation of RIPK1–RIPK3 necrosomes and thereby inhibits necroptosis [39]. The upregulation of A20 observed in our septic adults may reflect a compensatory anti-inflammatory feedback mechanism aimed at limiting necroinflammatory signaling [40].

While A20 dynamics are relatively well-characterized in autoimmune and chronic inflammatory conditions, their role in acute sepsis is less explored [41]. Our findings support the concept that sepsis-induced A20 elevation may serve as an endogenous attempt to restrain unchecked necroptosis and inflammation, particularly under conditions of metabolic stress indicated by hyperlactatemia.

### 4.4. Differences Across Age Groups and Disease States

Although adult patients in our cohort exhibited significantly higher RIPK-1 levels than pediatric patients, the distribution of this biomarker across diagnostic categories was remarkably consistent—decreasing progressively through sepsis, SIRS, cardiac cases, and healthy controls in both age groups. Pediatric patients demonstrated higher caspase-8 levels overall; however, within the sepsis group, caspase-8 was suppressed compared to healthy controls in both adults and children. This suggests that while age influences basal expression, the diagnostic patterns of necroptosis pathway activation are parallel in both cohorts.

Age-related divergence in programmed cell-death regulation has been previously reported. In murine models, T-cell-specific RIPK1 deficiency leads to activation of RIPK3, caspase-8, and mTORC1, driving T-cell senescence and age-related inflammatory phenotypes—an effect modulated by environmental signals [42]. This supports developmental differences in immune signaling pathways that may explain higher RIPK1 in adults and elevated caspase-8 in children [43,44].

Interestingly, among cardiac patients, caspase-8 levels were lower in adults than in children, potentially reflecting chronic inflammatory states. Estrogen deficiency and pre-menopausal hormonal changes exacerbate necroptosis-mediated cardiac injury through RIPK1-signaling in experimental models [45]. IL-1β—and to a lesser extent IL-18—was elevated in adult cardiac cases, suggesting partial necroptosis activation during sterile cardiac stress, as has been previously implicated in myocardial infarction and ischemia–reperfusion injury [46,47].

Despite these biochemical divergences, clinical severity scores and outcomes were comparable between adults and children, with outcome hardpoints demonstrating significant correlations with necroptosis biomolecules in both groups, underscoring the shared underlying biology of critical illness [48,49,50]. Our findings thus emphasize consistent pathobiological mechanisms across the lifespan, mediated by necroptosis and inflammatory cascades, despite developmental differences in baseline biomarker expression.

### 4.5. Prognostic and Therapeutic Implications

Our ROC analysis revealed that RIPK-1 and IL-18 demonstrated good diagnostic accuracy for sepsis within 24 h of ICU admission—suggesting strong potential as early biomarkers of disease severity. In contrast, caspase-8 showed an inverse predictive pattern, consistent with its anti-necroptotic, potentially protective role in this context. These findings collectively propose that necroptosis-related proteins may serve not only as indicators of severity but also as early therapeutic targets.

Importantly, in preclinical sepsis models, small-molecule RIPK1 inhibitors, such as necrostatins, have shown promising protective effects: Necrostatin-1 blocks RIPK1 kinase activity, limiting necrosome formation and downstream inflammation in experimental models of systemic inflammation and lung or kidney injury [51,52,53,54]. These data support the translational plausibility of targeting necroptosis in sepsis and underline the need for clinical research to explore RIPK1 inhibition in critically ill patients [55].

### 4.6. Strengths and Limitations

A major strength of this study lies in its prospective design and inclusion of both pediatric and adult cohorts, which allows for a comparative analysis of age-related differences in necroptosis pathways. Additionally, the use of multiple biomarkers related to both necroptosis, apoptosis, and inflammation (RIPK1, RIPK3, MLKL, caspase-8, IL-1β, IL-18, and A20) provides a multidimensional view of programmed cell death in critical illness. The application of ROC analysis also adds a translational aspect, supporting the diagnostic and prognostic relevance of these molecules.

However, several limitations must be acknowledged. First, although the overall sample size was sufficient to detect significant trends, subgroup analyses stratified by age and diagnostic category may have been underpowered, particularly within pediatric strata. Given the absence of statistically significant differences in RIPK3, MLKL, A20, IL-1β, or IL-18 between adults and children, and the lack of age-related effect modification in biomarker–outcome correlations, biomarker data were pooled in the primary analyses to preserve statistical power. This decision is further supported by the consistent direction and magnitude of diagnostic group differences across age groups. Nonetheless, age-specific comparisons are transparently reported in Table S1 and Figure 1, Figure 2 and Figure 3 for reference. Future studies with larger, stratified cohorts are warranted to validate these age-related findings more definitively. Second, while protein expression was quantified, functional assays to directly confirm necroptotic activity were not performed, which would strengthen mechanistic inferences. Third, the observational nature of the study precludes causal interpretations and may be influenced by unmeasured confounders such as co-morbidities, medications, or genetic factors influencing cell death pathways. Finally, serial measurements over time were not included, which could provide insight into dynamic changes in necroptosis signaling during the course of illness and recovery.

### 4.7. Perspective and Future Directions

This study advances our understanding of the immunopathology of sepsis by identifying necroptosis as a key contributor to disease progression and outcome. The identical trends between age groups and the differential expression of necroptosis-related markers among disease states not only enrich our biological insight but also open the door to personalized approaches in critical care. Future studies should explore longitudinal trajectories of necroptosis biomarkers, validate their predictive value in larger, multicenter cohorts, and assess their response to targeted therapies. Interventional trials using RIPK1 inhibitors or caspase-8 modulators could test the therapeutic potential of modulating cell death pathways in sepsis. Additionally, integrating these biomarkers into clinical decision-support tools may enhance early risk stratification and treatment allocation.

In conclusion, the findings from this study provide a strong rationale for considering necroptosis not merely as a biological consequence but as a potential driver of systemic inflammation and organ dysfunction in sepsis. Translating these insights into bedside applications represents a promising frontier for improving outcomes in both pediatric and adult critical care populations.

## 5. Conclusions

This study underscores the pivotal role of necroptosis in the pathophysiology of sepsis, as reflected by elevated levels of RIPK1, IL-1β, and IL-18 and reduced expression of caspase-8 across both pediatric and adult patients. These molecular alterations were associated with increased disease severity and prolonged ICU stay, particularly in septic individuals. The observed upregulation of A20 may represent a compensatory anti-necroinflammatory mechanism. Although the study was not primarily designed for age-stratified comparisons, the inclusion of both age groups allowed for exploratory analysis, revealing consistent biomarker expression patterns across the lifespan. Collectively, these findings support the translational potential of necroptosis-related biomarkers for risk stratification and therapeutic targeting in critically ill patients of all ages.

## Figures and Tables

**Table 1:** Subject Characteristics and Diagnostic Stratification, Including Healthy Controls.

Subject Characteristics	Total (n = 88)	Adults (n = 56)	Children (n = 32)	*p*-Value
Gender, n (%)				0.825
Male	62 (70.5)	39 (69.6)	23 (71.9)	
Female	26 (29.5)	17 (30.4)	9 (28.1)	
Age (years), mean ± SD	33.1 ± 25	47.4 ± 21	8.1 ± 5.5	<0.001
Body weight (kg), mean ± SD	56.8 ± 33	70.6 ± 28	32.5 ± 24	<0.001
BMI (kg/m^2^), mean ± SD	23.3 ± 4.9	25.5 ± 4.3	19.4 ± 2.9	<0.001
BMI Nutritional Status, n (%)				0.020
Undernutrition	8 (9.1)	2 (3.6)	6 (18.8)	
Normal weight	55 (62.5)	33 (58.9)	22 (68.8)	
Overweight	18 (20.5)	15 (26.8)	3 (9.4)	
Obesity	7 (8.0)	6 (10.7)	1 (3.1)	
Study Group, n (%)				0.133
Sepsis/Septic Shock	23 (26.1)	17 (30.4)	6 (18.8)	
SIRS	29 (33.0)	15 (26.8)	14 (43.8)	
Cardiac	19 (21.6)	15 (26.8)	4 (12.5)	
Healthy Controls	17 (19.3)	9 (16.1)	8 (25.0)	

Abbreviations: SIRS = Systemic Inflammatory Response Syndrome; BMI = Body Mass Index.

**Table 2:** Clinical Characteristics of the Study Population (Excluding Healthy Controls).

Patients Characteristics	Total (n = 71)	Adults (n = 47)	Children (n = 24)	*p*-Value
Temperature (°C), mean ± SD	37.9 ± 0.7	37.7 ± 0.6	38.1 ± 0.9	0.072
APACHE II, mean ± SD	14.9 ± 9.7	16.2 ± 11	12.6 ± 6.9	0.147
SOFA score ≥ 2, n (%)	44 (62)	26 (55.3)	18 (75.0)	0.106
Comorbidities, n (%)	31 (35.2)	29 (51.8)	2 (6.3)	<0.001
Primary Clinical Diagnoses				0.007
Pneumonia	17 (19.3)	9 (16.1)	8 (25.0)	
ARDS	8 (9.1)	1 (1.8)	7 (21.9)	
Myocarditis	12 (13.6)	8 (14.3)	4 (12.5)	
Various Infections	24 (27.3)	16 (28.6)	9 (27.0)	
Traumatic Brain Injury	6 (6.8)	3 (5.4)	3 (9.4)	
Trauma/Surgery	10 (11.4)	9 (16.1)	1 (3.1)	
Therapeutic Interventions, n (%)				
Mechanical ventilation	37 (52.1)	26 (55.3)	11 (45.8)	0.449
Vasoactive agents	49 (69.0)	40 (85.1)	9 (37.5)	<0.001
ICU stay (days), mean ± SD	7.14 ± 7.5	7.37 ± 5.6	6.75 ± 9.9	0.712
Duration of mechanical ventilation (days)	5.22 ± 7.2	4.20 ± 1.7	6.75 ± 11	0.306
Duration of vasoactive therapy (days)	4.1 ± 1.7	4.0 ± 1.7	6.9 ± 11	0.771
Mortality, n (%)	2 (2.8)	1 (2.1)	1 (4.2)	0.623

Abbreviations: ARDS = Acute Respiratory Distress Syndrome; APACHE II = Acute Physiology and Chronic Health Evaluation Score II; SOFA = Sequential Organ Failure Assessment.

**Table 3:** Differences in Key Characteristics Between Study Groups.

Variable	Sepsis	SIRS	Cardiac	Healthy	*p*-Value
Participants, n (%)	23 (26.1)	29 (33.0)	19 (21.6)	17 (19.3)	—
Age (years), mean ± SD	47 ± 30	29 ± 26	32 ± 21	23 ± 12	0.012
Body weight (kg), mean ± SD	52 ± 32	55 ± 37	77 ± 24	43 ± 24	0.011
BMI (kg/m^2^), mean ± SD	23 ± 4.6	23 ± 6.5	25 ± 4.2	22 ± 1.7	0.421
ICU length of stay (days), mean ± SD	9.7 ± 5.5	9.8 ± 9.6	5.5 ± 4.0	—	0.098

Abbreviations: SIRS = Systemic Inflammatory Response Syndrome; BMI = Body Mass Index; ICU = Intensive Care Unit.

**Table 4:** Biomarkers of Necroptosis, Apoptosis, and Inflammatory Pathway Activation Across Diagnostic Groups.

Biomolecules All Subjects	Sepsis (n = 23)	SIRS (n = 29)	Cardiac (n = 19)	Healthy (n = 17)	*p*-Value **
RIPK-1 (ng/mL), median (IQR)	30.3 (16–47) *	8.9 (4.6–22) ^#^	3.7 (2.1–6.1) *	2.5 (1.7–3.5) *^,#^	<0.001
RIPK-3 (ng/mL), median (IQR)	3.8 (1.2–7.0)	1.9 (0.8–2.9)	1.1 (0.6–2.7)	1.5 (0.7–2.2)	0.085
MLKL (ng/mL), median (IQR)	6.5 (5.0–7.0)	6.6 (4.3–7.6)	6.0 (3.8–6.3)	5.2 (4.7–6.0)	0.131
A20 (ng/mL), median (IQR)	0.72 (0.5–0.9) *	0.55 (0.4–0.7)	0.52 (0.5–0.6)	0.37 (0.3–0.5) *	0.065
IL-1β (pg/mL), median (IQR)	561 (387–948) *	723 (406–940) ^#^	933 (658–1027) ^^^	84 (70–99) *^,#,^^	<0.001
IL-18 (pg/mL), median (IQR)	9815 (7220–13,504) *	9033 (3671–12,588) ^#^	5417 (3133–6990) ^^^	147 (132–206) *^,#,^^	<0.001
Caspase-8 (ng/mL), median (IQR)	13.8 (12–15) *	17.4 (15–27)	16.6 (8.9–54)	24.6 (16–34) *	0.015

Abbreviations: SIRS = Systemic Inflammatory Response Syndrome; RIPK = Receptor-Interacting Protein Kinase; MLKL = Mixed Lineage Kinase Domain-Like Protein; A20 = TNFAIP3 ubiquitin-editing enzyme. ** Independent-Samples Kruskal–Wallis Test. Pairwise Comparisons adjusted by Bonferroni correction for multiple tests between diagnostic study groups (*p* < 0.05) and sepsis *, SIRS ^#^, or cardiac patients ^.

## Data Availability

Clinical data presented in this study are all contained within this article. The institution’s rights, including legal and ethical concerns, patient privacy, and confidentiality, restrict access to detailed data sharing.

## Supplementary Materials

The following supporting information can be downloaded at: https://www.mdpi.com/article/10.3390/biomedicines13071747/s1. Table S1: Laboratory Data Within the First 24 Hours of ICU Admission; Table S2: Biomarkers of Necroptosis, Apoptosis, and Inflammatory Pathway Activation Across Diagnostic Groups Stratified by Age Group; Figure S1: A. Scatterplot showing the correlation between Caspase-8 levels and ICU length of stay in patients with sepsis or SIRS and cardiac patients compared to the healthy control group. B. Scatterplot showing the correlation between RIPK-1 levels and ICU length of stay in pediatric and adult patient groups.

## Abbreviations

The following abbreviations are used in this manuscript:

ICU	Intensive Care Unit
ARDS	Acute Respiratory Distress Syndrome
IL	Interleukin
SIRS	Systemic Inflammatory Response Syndrome
ELISA	Enzyme-linked Immunosorbent Assay
APACHE	Acute Physiology and Chronic Health Evaluation Score II
SOFA	Sequential Organ Failure Assessment
BMI	Body Mass Index
RIPK	Receptor-Interacting Protein Kinase
MLKL	Mixed Lineage Kinase Domain-Like Protein
PeLOD	Pediatric Logistic Organ Dysfunction
PRISM	Pediatric Risk of Mortality
qSOFA	quick Sepsis-Related Organ Failure Assessment
ROC	Receiver Operating Characteristic
mTORC1	Mammalian Target of Rapamycin Complex 1
