# Supplementary material for: Necroptotic and Apoptotic Pathways in Sepsis: A Comparative Analysis of Pediatric and Adult ICU Patients
Source: Biomedicines. 2025 Jul 17;13(7):1747. doi: 10.3390/biomedicines13071747 (PMC12292671; doi:10.3390/biomedicines13071747)
Supplement: Supplementary file 1 [file biomedicines-13-01747-s001.zip › biomedicines-3702367-supplementary.pdf]

Necroptotic and Apoptotic Pathways in Sepsis: A Comparative Analysis of Pediatric and Adult ICU Patients

Supplementary Materials

Table S1. Laboratory Data Within the First 24 Hours of ICU Admission

Parameter	Total (n = 88)	Adults (n = 56)	Children (n = 32)	p-value*
Participants, n (%)	88 (100)	56 (63.6)	32 (36.4)	—
pH, mean ± SD	7.35 ± 0.4	7.34 ± 0.6	7.39 ± 0.1	0.589
PaO_2 (mmHg), mean ± SD	104 ± 38	102 ± 31	109 ± 48	0.418
$PaCO_2$ (mmHg), mean ± SD	37.2 ± 7.7	37.8 ± 8.5	35.9 ± 6.2	0.369
HCO_3^- (mmol/L), mean ± SD	22.3 ± 4.0	22.9 ± 4.3	21.0 ± 3.2	0.068
WBC ($\times 10^3/\mu L$), mean ± SD	13.5 ± 9.0	14.1 ± 10.4	12.5 ± 5.5	0.476
Positive blood culture, n (%)	4 (5.7)	3 (6.4)	1 (1.4)	0.730
CRP (mg/dL), mean ± SD	14.2 ± 14.0	14.6 ± 14.0	13.3 ± 14.0	0.731
Procalcitonin (ng/mL), mean ± SD	0.7 ± 3.5	0.2 ± 0.1	1.8 ± 6.1	0.074
Lactate (mg/dL), mean ± SD	10.3 ± 15.0	11.4 ± 17.0	8.0 ± 7.7	0.357
Glucose (mg/dL), mean ± SD	135 ± 61	152 ± 68	103 ± 25	<0.001
Urea (mg/dL), mean ± SD	39 ± 33	49 ± 36	20 ± 15	<0.001
Creatinine (mg/dL), mean ± SD	1.95 ± 6.2	2.5 ± 7.6	0.9 ± 0.9	0.300
SGOT (U/L), mean ± SD	85 ± 159	98 ± 189	59 ± 67	0.329
SGPT (U/L), mean ± SD	56 ± 185	74 ± 226	21 ± 13	0.257
Bilirubin (mg/dL), mean ± SD	1.2 ± 1.0	1.2 ± 1.0	0.5 ± 0.4	0.008
High-sensitivity Troponin I (pg/mL), mean ± SD	4755 ± 14489	5023 ± 15089	1597 ± 2654	0.654
RIPK-1 (ng/mL), median (IQR)	8.23 (3.6-28.6)	20.2 (7.4-46.5)	6.2 (4.4-16.2)	0.021
RIPK-3 (ng/mL), median (IQR)	1.96 (0.7-3.8)	2.05 (0.8-3.9)	2.51 (0.8-4.9)	0.548
MLKL (ng/mL), median (IQR)	6.01 (4.7-6.8)	6.67 (4.8-7.6)	5.96 (4.3-6.6)	0.118
A20 (ng/mL), median (IQR)	0.55 (0.5-0.7)	0.55 (0.4-0.9)	0.59 (0.5-0.7)	0.738
IL-1β (pg/mL), median (IQR)	540 (247-940)	636 (420-1172)	694 (531-994)	0.956
IL-18 (pg/mL), median (IQR)	8120 (3159-10958)	8828 (5387-12249)	8675 (3468-10854)	0.457
Caspase-8 (ng/mL), median (IQR)	16.4 (13-28)	13.8 (11-20)	19.1 (14-28)	0.008

Abbreviations: CRP = C-reactive protein; WBC = white blood cell count; SGOT = serum glutamic-oxaloacetic transaminase; SGPT = serum glutamic-pyruvic transaminase; HS TnI = high-sensitivity troponin I.

* *t-tests or one-way ANOVA for normally distributed data, and Mann–Whitney U or Kruskal–Wallis tests for non-normally distributed data as appropriate.*

Table S2. Biomarkers of Necroptosis, Apoptosis, and Inflammatory Pathway Activation Across Diagnostic Groups Stratified by Age Group.

Biomolecules Adults	Sepsis (n = 17)	SIRS (n = 15)	Cardiac (n = 15)	Healthy (n = 9)	p-value**
RIPK-1 (ng/mL), median (IQR)	35.6 (19–49)*	11.6 (4.7–33)*,#	4.49 (2.2–8.6)*	3.17 (1.6–6.9)*,#	<0.001
RIPK-3 (ng/mL), median (IQR)	2.91 (1.1–7.4)*	0.83 (0.4–2.1)*	2.28 (0.6–2.8)	1.7 (0.3–2.4)	0.124
MLKL (ng/mL), median (IQR)	6.72 (5.3–7.7)*	6.87 (6.1–8.2)#	3.81 (1.9–6.2)*,#	4.73 (3.5–5.2)*,#	0.002
A20 (ng/mL), median (IQR)	0.57 (0.5–0.9)*	0.52 (0.4–0.8)	0.55 (0.5–0.8)	0.37 (0.3–0.5)*	0.049
IL-1β (pg/mL), median (IQR)	480 (326–1111)*	596 (406–1172)#	937 (504–1357)^	84 (67–103)*,#,^	<0.001

IL-18 (pg/mL), median (IQR)	9938 (7099–13679)*	8071 (4654–12136)#	5613 (4783–9229)^	147 (122–231)*,#,^	<0.001
Caspase-8 (ng/mL), median (IQR)	13.8 (12–18)*	16.2 (12–25)	11.8 (8.2–33)^	24.6 (15–36)*,^	0.098

Biomolecules Children	Sepsis (n = 6)	SIRS (n = 14)	Cardiac (n = 4)	Healthy (n = 8)	p-value**
RIPK-1 (ng/mL), median (IQR)	15.9 (10–29)*	5.45 (4.6–14)#,^	2.16 (2.1–3.3)*,^	2.46 (1.7–2.9)*,#	<0.001
RIPK-3 (ng/mL), median (IQR)	3.86 (3.7–6.7)	2.29 (1.3–4.8)	0.61 (0.5–7.9)	1.24 (0.7–1.9)	0.245
MLKL (ng/mL), median (IQR)	4.74 (4.7–5.5)	5.52 (4.1–7.4)	6.12 (6.0–6.3)	6.07 (5.6–6.6)	0.542
A20 (ng/mL), median (IQR)	0.72 (0.6–0.7)	0.58 (0.5–0.6)	0.51 (0.5–0.9)	0.54 (0.5–0.7)	0.278
IL-1β (pg/mL), median (IQR)	795 (5779–10204)*	722 (453–915)#	574 (559–783)^	14.3 (8.8–52)*,#,^	<0.001
IL-18 (pg/mL), median (IQR)	8489 (7139–13621)	9102 (3468–12123)	3028 (2398–5936)	-	0.272
Caspase-8 (ng/mL), median (IQR)	12.8 (12–21)*	20.6 (16–29)*	32.9 (22–54)*	24.6 (19–41)*	0.023

Abbreviations: SIRS = Systemic Inflammatory Response Syndrome; RIPK = Receptor-Interacting Protein Kinase; MLKL = Mixed Lineage Kinase Domain-Like Protein; A20 = TNFAIP3 ubiquitin-editing enzyme.
**Independent-Samples Kruskal-Wallis Test (2-sided tests, p<0.05).
Pairwise Comparisons adjusted by the Bonferroni correction for multiple tests (p<0.05) between diagnostic study groups and sepsis*, SIRS# or cardiac patients^

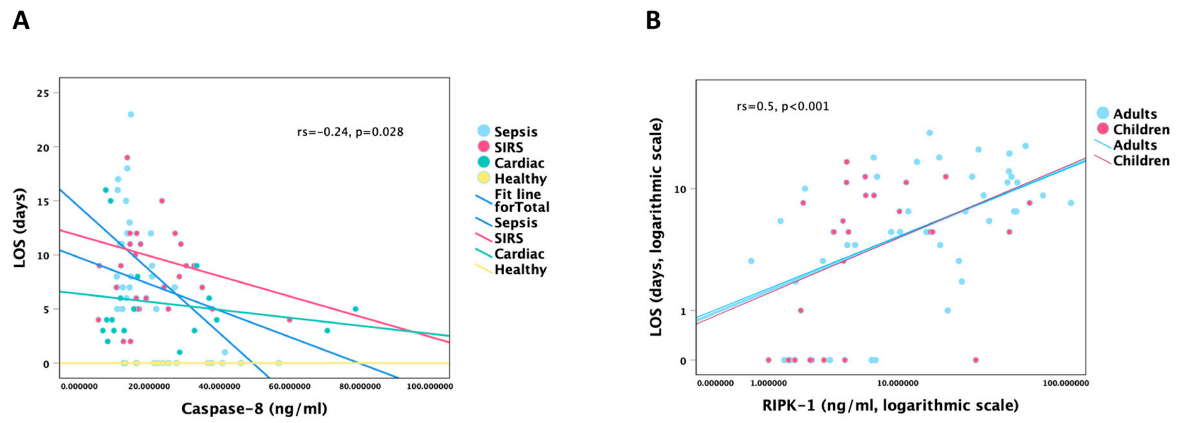

Figure S1. **A.** Scatterplot showing the correlation between Caspase-8 levels and ICU length of stay in patients with sepsis or SIRS, and cardiac patients, compared to the healthy control group. **B.** Scatterplot showing the correlation between RIPK-1 levels and ICU length of stay in pediatric and adult patient groups.

Abbreviations: SIRS = Systemic Inflammatory Response Syndrome; RIPK = Receptor-Interacting Protein Kinase; LOS = Length Of Stay.
